# Distinct Roles of Met and Interacting Proteins on the Expressions of *takeout* Family Genes in Brown Planthopper

**DOI:** 10.3389/fphys.2017.00100

**Published:** 2017-02-21

**Authors:** Xinda Lin, Ling Zhang, Yanyun Jiang

**Affiliations:** Department of Biology, College of Life Sciences, China Jiliang UniversityHangzhou, China

**Keywords:** brown planthopper, juvenile hormone, Met, Taiman, ß-Ftz-F1, *takeout*

## Abstract

The *takeout* family genes encode relatively small proteins that are related to olfaction and are regulated by juvenile hormone (JH). The *takeout* genes modulate various physiological processes, such as behavioral plasticity in the migratory locust *Locusta migraloria* and feeding and courtship behaviors in *Drosophila*. Therefore, to understand the regulatory mechanism of these physiological processes, it is important to study the expressions of the *takeout* genes that are regulated by JH signaling. We used quantitative real-time PCR (qRTPCR) to study the role of JH signaling in the regulation of the *takeout* family genes in the brown planthopper *Nilaparvata lugens* (*N. lugens*) through the application of Juvenile hormone III (JHIII) and the down-regulation of key genes in the JH signaling pathway. The topical application of JHIII induced the expressions of most of the *takeout* family genes, and their expressions decreased 2 and 3 days after the JHIII application. Down-regulating the brown planthopper JH receptor NlMethoprene-tolerant (NlMet) and its interacting partners, NlTaiman (NlTai) and Nlß-Ftz-F1 (Nlß-Ftz), through RNAi, exhibited distinct effects on the expressions of the *takeout* family genes. The down-regulation of *NlMet* and *NlKrüppel-homolog 1* (*NlKr-h1*) increased the expressions of the *takeout* family genes, while the down-regulation of the Met interacting partners *NlTai* and *Nlß-Ftz* decreased the expressions of most of the *takeout* family genes. This work advanced our understanding of the molecular function and the regulatory mechanism of JH signaling.

## Introduction

The *takeout* family genes encode relatively small proteins that are related to olfaction (Dauwalder et al., 2002; Saito et al., 2006; Hagai et al., 2007). Since the first characterization of the *takeout* gene in *Drosophila melanogaster* (Fujikawa et al., 2006), homologs of *takeout* have been identified from a broad range of insect species, including *Phormia regina* (Fujikawa et al., 2006), *Manduca sexta* (Du et al., 2003), *Bombyx mori* (Saito et al., 2006), *Apis mellifera* (Hagai et al., 2007), *Reticulitermes flavipes* (Dauwalder et al., 2002), and *Locusta migraloria* (Guo et al., 2011). The migratory locust *Locusta migraloria takeout* modulates behavioral plasticity (Guo et al., 2011), i.e., the switch between attraction and repulsion during the phase transition (Guo et al., 2011). The *takeout* gene was found to be regulated by the circadian rhythm and affects feeding behavior (So et al., 2000; Meunier et al., 2007), locomotion (Meunier et al., 2007), and male courtship behavior (Dauwalder et al., 2002) in *D. melanogaster*. *Takeout* is also involved in the trail-following behavior of the termite *Reticulitermes flavipes* (Dauwalder et al., 2002). The expressions of the *takeout* genes are usually male biased (Hagai et al., 2007; Vanaphan et al., 2012) and are regulated by age and nutrition (Du et al., 2003; Hagai et al., 2007). A circadian transcription factor PAR domain protein 1 (Pdp1ε) mediated the regulation of *takeout* by the circadian rhythm (Dauwalder et al., 2002). The expression of *takeout* is usually regulated by a crucial hormone in insects, Juvenile hormone (JH; Du et al., 2003; Hagai et al., 2007). However, the regulatory mechanism of *takeout* expression by JH remained unclear.

JH is secreted by the corpora allata (CA) and belongs to a type of sesquiterpenoid and regulates development, reproduction, polyphenism (a special case of phenotypic plasticity), and behaviors, such as feeding and mating (Jindra et al., 2013). The signal transduction pathway of JH is initiated by the release of the JH ligand, followed by binding to the intracellular receptor Methoprene-tolerant (Met; Bernardo and Dubrovsky, 2012; Jindra et al., 2013) through an interaction between Met and Taiman (Tai), which is an EcR coactivator (Zhu et al., 2006; Li et al., 2011, 2014), possibly also through an interaction between Met and ß-Ftz-F1 (Zhu et al., 2006; Yoo et al., 2011; Bernardo and Dubrovsky, 2012), leading to transcriptional changes of downstream genes and the regulation of developmental and physiological processes (Truman and Riddiford, 2002; Belles et al., 2005; Flatt et al., 2005). JH induced the transcription of *Kr-h1* through the binding of the Met-Tai complex to the E-Box at the 5′ of the *Kr-h1* gene in the mosquito *Aedes aegypti* (Zhu et al., 2010; Li et al., 2011, 2014). Works in *Tribolium* also indicated that the function of *Kr-h1* is dependent on the JH receptor Met (Minakuchi et al., 2009). Consistently, we previously showed that the brown planthopper *Kr-h1* is induced by JH or its mimics (Jin et al., 2014).

The brown planthopper, *Nilaparvata lugens* (*N. lugens*), which is one of the most important insect pests in rice production, exhibits polyphenism, and has the long wing and short wing forms. The long wing form is migratory, and the short wing form is reproductive. Previous studies have shown that the wing form of brown planthopper is regulated by JH and the density and developmental stage of the rice plant (Kisimoto, 1956, 1965; Iwanaga and Tojo, 1986; Ayoade et al., 1999; Bertuso et al., 2002). More recently, it was found that the wing form of the brown planthopper is regulated by two alternative receptors in the insulin signaling pathway and the JNK signaling pathway (Xu et al., 2015; Lin et al., 2016a,b). Interestingly, we found that wounding also affects the wing form through the regulation of the transcription factor Foxo (Lin et al., 2016c).

The regulation of target genes by JH signaling is bidirectional; certain genes are activated by JH, and other genes are repressed or not affected. The activation is mediated by the JH receptor Met (Schwinghammer et al., 2011), and the repression is mediated by Met through the recruitment of the Hairy/Goucho molecular system (Hagai et al., 2007). However, the role of Met and its interacting partners in regulating the expressions of the *takeout* genes remained unknown, and the role of the *takeout* genes in wing polyphenism remained unclear due to the lack of knowledge of behavior plasticity. Moreover, the complete identification of the *N. lugens* genome sequence (Xue et al., 2014) and key biological characteristics, such as migration and behavior plasticity, are important for pest control and predictions of pest outbreaks, making *N. lugens* an appropriate model for studying the role of gene families, such as the *takeout* family genes. Here, we use quantitative real-time PCR to study the role of JH signaling in the regulation of *N. lugens takeout* genes by the topical application of JH or the down-regulation of Met and its interacting partners through RNAi.

## Materials and methods

### Insects

The brown planthopper (*N. lugens*) insectary population was provided by Professor Zhu Zeng-Rong, Institute of Insect Sciences, Zhejiang University. The insects were cultured with rice seedling and raised at a temperature = 25°C, relative humidity = 60%, and a photoperiod = 16 L:8 D.

### Construction of phylogenetic trees and WebLogo conserved amino acid analysis

A Phylogenetic tree, including 17 brown planthopper Takeout proteins and 65 homologs of other species, was constructed, and the sequences were downloaded from the GenBank database (http://www.ncbi.nlm.nih.gov/genbank/). The phylogenetic tree was constructed by MEGA 6.0 software using the Neighbor-Joining method and a bootstrap value of 1000. The predicted amino acid sequences of 17 *N. lugens* Takeout proteins were aligned into WebLogo (http://weblogo.berkeley.edu/logo.cgi) and were compared in pairs using the default settings.

### JHIII treatment

The juvenile hormone III (JHIII, Sigma Aldrich, USA) was dissolved in acetone at a concentration of 1 μg/μL, with acetone as a control group, and a volume of 0.2 μL was applied to the back of each brown planthopper at the 5th nymph stage; the brown planthoppers were collected 1 or 3 days after the treatment and ground in TRIzol, and the total RNA was then extracted.

### RNA interference

The DNA fragments used for the dsRNA synthesis were amplified through PCR using *NlMet, NlKr-h1, NlTai*, and *Nl*β*-Ftz* cloned into PMD18-T separately as templates. The primers are listed in Table 1. Double-stranded RNA of *NlMet, NlKr-h1, NlTai*, and *Nl*β*-Ftz* were synthesized using the RNA Production System-T7 kit (RiboMAX Large Scale, Promega). dsGFP was used as a control. The 5th instar nymphs of *N. lugens* were injected. The Narishige Injection System (MN-151, Narishige) was used for the dsRNA injection. One or three days after the injection, the insects were collected for RNA extraction.

**Table 1:** **Primers for dsRNA synthesis**.

**Name**	**Nucleotide sequence (5′–3′)**
dsGFPT7F	GGATCCTAATACGACTCACTATAGGAAGGGCGAGGAGCTGTTCACCG
dsGFPT7R	GGATCCTAATACGACTCACTATAGGCAGCAGGACCATGTGATCGCGC
dsNlTaiF	TAATACGACTCACTATAGGGAGACCACTTCATTCATTCAGGCTCGGC
dsNlTaiR	TAATACGACTCACTATAGGGAGACCACCCACTCACACTACCACCACT
dsNlβ-FtzF	TAATACGACTCACTATAGGGAGACCACCGACCAGATCTCGTTGCTGA
dsNlβ-FtzR	TAATACGACTCACTATAGGGAGACCACGCAGCCACAAGTAGAATCCG
dsNlMetT7F	TAATACGACTCACTATAGGGAGACCACCAACCAGCAGATGAACCTGA
dsNlMetT7R	TAATACGACTCACTATAGGGAGACCACGCAAAGCCTCGTACTCTTGG
dsNlKrhT7F	TAATACGACTCACTATAGGGAGACCACGTGGGGTTCAGTCCTGAGGA
dsNlKrhT7R	TAATACGACTCACTATAGGGAGACCACCAGTCGAACACACACCGGAG

### RNA extraction and quantitative real-time PCR

Total RNA was extracted using the TRIzol RNA extraction kit (TaKaRa). Reverse transcription was carried out using the First Strand cDNA Synthesis kit (Roche). The real-time quantitative PCR kit SuperReal PreMix (SYBR Green, Tiagen, Beijing) was used. All primers were synthesized by Sangon (Shanghai). All primers are listed in Table 2. The reference genes were selected based on previous reports (Yuan et al., 2014).

**Table 2:** **Primers for Quantitative PCR**.

**Gene**	**Forward**	**Reverse**
*RPS15*	TAAAAATGGCAGACGAAGAGCCCAA	TTCCACGGTTGAAACGTCTGCG
*actQ*	TGGACTTCGAGCAGGAAATGG	ACGTCGCACTTCATGATCGAG
*NlTO1*	CAATGGCTCATCATCACTCA	GGGAATGGCTATTCTTCCAT
*NlTO2*	GCCAATGATGCAAAGGATAC	ATGCAGTCTTCGAGTTTTGC
*NlTO3*	GCCGTCAATTACAAGGCTAA	ATTTGCAGCTTGTTCAGGTC
*NlTO4*	CACCAGAGGGTTCTCAGCTA	ACAATACGGGGCACATAGAA
*NlTO5*	GGTCAGCAGGCTATACCAAA	TCTGGTGCCCTGGTTTACTA
*NlTO6*	TTCGAACCCCTCTACATTGA	GTATTGCTTGGTCCATGAGC
*NlTO7*	GACTGTCCAAGTCCCATGTC	TGTACATGCCCTTGATGTTG
*NlTO8*	AGCTATTCCTTCCCTGCATT	AGTAGCATTGGCTTTCATGG
*NlTO9*	AACGGCCGAGCTTACTTCAA	CACCTCCTTCCAGTTCTCGT
*NlTO10*	CACATCATGAAGAGTGCGCT	CTCTCGGGCATGGTTTGATG
*NlTO11*	CCAATCCAAGGAGAGGGTGA	GAGTCTTGCCGTTCTTCACC
*NlTO12*	CTGAATTTGACGCCGGGTAG	GATGAGCCATTGATGAGGCA
*NlTO13*	TGGTGATTTGAGCGAGCCTA	GGGTGAGCTTGCATTTTCCA
*NlTO14*	GTTCTGGGGCATAGACGACT	TCATCGCATCTCCCAGTTGT
*NlTO15*	CGGACTCCAGGATGTTGACT	TAGCATCCCCTTGTCCTGTG
*NlTO16*	TGGAACAGGGCCTAGTGATG	CGCCATTGAAGAGATCTCCC
*NlTO17*	ATCGTTGGCCTTGAATCACG	CCTTCGCCGAATATTGGCAA
*NlMet*	GGTGGTAAACGGATTGGAAA	CATCGTCAGCCAACTCGATA
*NlKr-h1*	TGATGAGGCACACGATGACT	ATGGAAGGCCACATCAAGAG
*NlTai*	ATGATCCCAACCACTTCAGC	TTCCACTCACACTACCACCA
*Nlβ-Ftz*	CCATGAGAACCCGTAATCCG	CACACTCGAGTCCCTTGATG

The RNA concentration was measured using NanoDrop 1000 (Thermo, USA). The primers were designed in the range of 90–110 bp for the qRT-PCR measurement of the *NlTO* genes. Three replicates were used for the qRT-PCR reactions of each sample. In total, a 20 μL reaction was used for the qRT-PCR reaction, including 10 μL 2 × SuperReal PreMix, 0.6 μL upstream and downstream primers (10 μmol • L^−1^), 0.6 μL 50 × ROX Reference Dye, 2 μL cDNA template, and 6.2 μL DEPC-treated water. Using a two-step qRT-PCR amplification procedure, the pre-denaturation was as follows: 95°C 1 min, 1 cycle; The qRT-PCR reactions were as follows: 95°C 3 s, 58°C 30 s, 40 cycles. All data were analyzed using the 2^−ΔΔCt^ method (Livak and Schmittgen, 2001).

### Statistics and heatmap

SPSS 20.0 was used for the data analysis. For the analysis of the qRT-PCR experiment, student's *t*-test was used. A heatmap was constructed using HemI1.03, and the fold changes of relative expressions were logarithmically transformed. The clustering method was a hierarchical average linkage, and the similarity metric was the Pearson distance.

## Results

### Cloning and analysis of the brown planthopper *takeout* family genes

**Figure 1:**
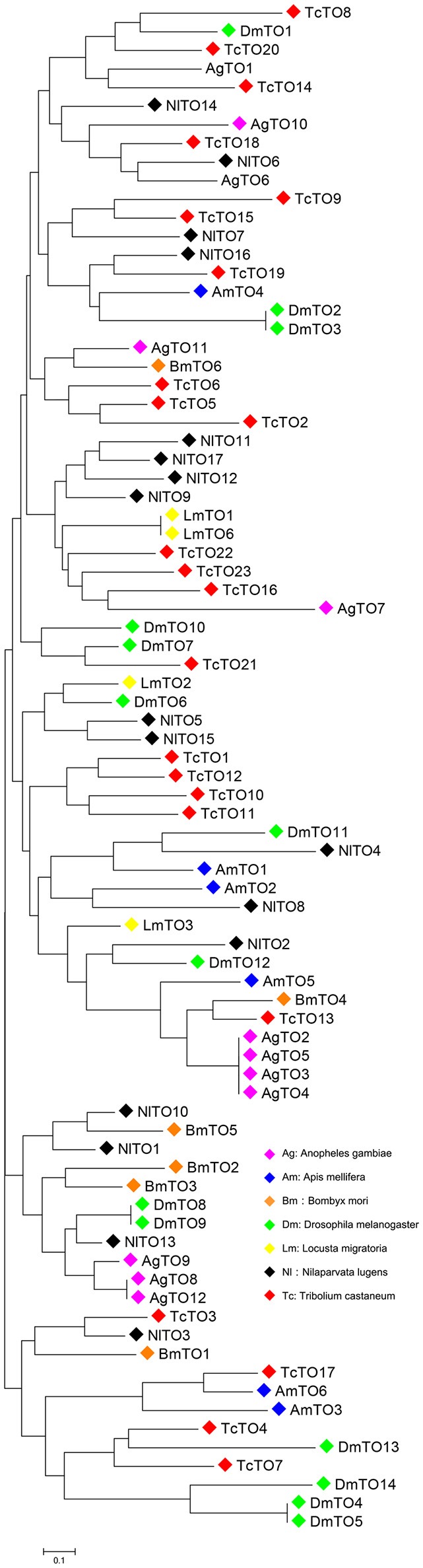
**Phylogenetic analysis of Takeout family proteins from *N. lugens* and other species**. AgTO1 (XM_311285), AgTO2 (XM_321081), AgTO3 (XM_003435936), AgTO4 (XM_003435937), AgTO5 (XM_003435938), AgTO6 (XM_321079), AgTO7 (XM_321075), AgTO8 (XM_313180), AgTO9 (XM_313181), AgTO10 (XM_001230706), AgTO11 (XM_309482), AgTO12 (XM_307380), AmTO1 (GB48492-PA), AmTO2 (GB42798-PA), AmTO3 (GB42796-PA), AmTO4 (GB42799-PA), AmTO5 (GB42800-PA), AmTO6 (GB42704-PA), BmTO1 (XP_004927145), BmTO2 (NP_001036949), BmTO3 (NP_001036945), BmTO4 (XP_004923014), BmTO5 (XP_004932669), BmTO6 (XP_012548133), DmTO1 (FBpp0078169), DmTO2 (FBpp0082691), DmTO3 (FBpp0307590), DmTO4 (FBpp0083445), DmTO5 (FBpp0311940), DmTO6 (FBpp0084027), DmTO7 (FBpp0084184), DmTO8 (FBpp0084185), DmTO9 (FBpp0308365), DmTO10 (FBpp0308366), DmTO11 (FBpp0084473), DmTO12 (FBpp0084474), DmTO13 (FBpp0290041), DmTO14 (FBpp0083446), LmTO1 (GU722575), LmTO2 (CO856064), LmTO3 (CO825835), LmTO6 (KM503135), TcTO1 (XP_967109), TcTO2 (EFA05096), TcTO3 (EFA05095), TcTO4 (XP_966559), TcTO5 (XP_974592), TcTO6 (XP_974610), TcTO7 (XP_966559), TcTO8 (XP_008190426), TcTO9 (EFA05633), TcTO10 (XP_970866), TcTO11 (XP_970866), TcTO12 (EFA05635), TcTO13 (KYB27715), TcTO14 (XP_973361), TcTO15 (EFA03576), TcTO16 (EFA03557), TcTO17 (XP_015836023), TcTO18 (XP_972960), TcTO19 (XP_972997), TcTO20 (XP_001812695), TcTO21 (XP_015840904), TcTO22 (EEZ98654), TcTO23 (XP_974890). Ag, *Anopheles gambia*; Am, *Apis mellifera*; Bm, *Bombyx mori*; Dm, *Drosophila melanogaster*; Lm, *Locusta migraloria*; Tc, *Tribolium castaneum*.

We searched the brown planthopper *N. lugens* genome (Xue et al., 2014) and InsectBase (Yin et al., 2016). We identified 17 *takeout* homologs. We then cloned, sequenced and named all *takeout* homologs-*takeout 1* (*TO1*) to *takeout 17* (*TO17*). The phylogenetic tree analysis showed that the brown planthopper *takeout* genes are conserved across the species (Figure 1). NlTO11 clustered with NlTO17, and both clustered with NlTO7 (Figure 1). These three Takeout homologs together clustered with NlTO12 and TcTO22 (Figure 1). NlTO15 clustered with NlTO5, and both clustered together with NlTO14 (Figure 1). Four homologs, including NlTO3, NlTO4, NlTO6, and NlTO13, are relatively distant, and each has close homologs from other species (Figure 1).

**Figure 2:**
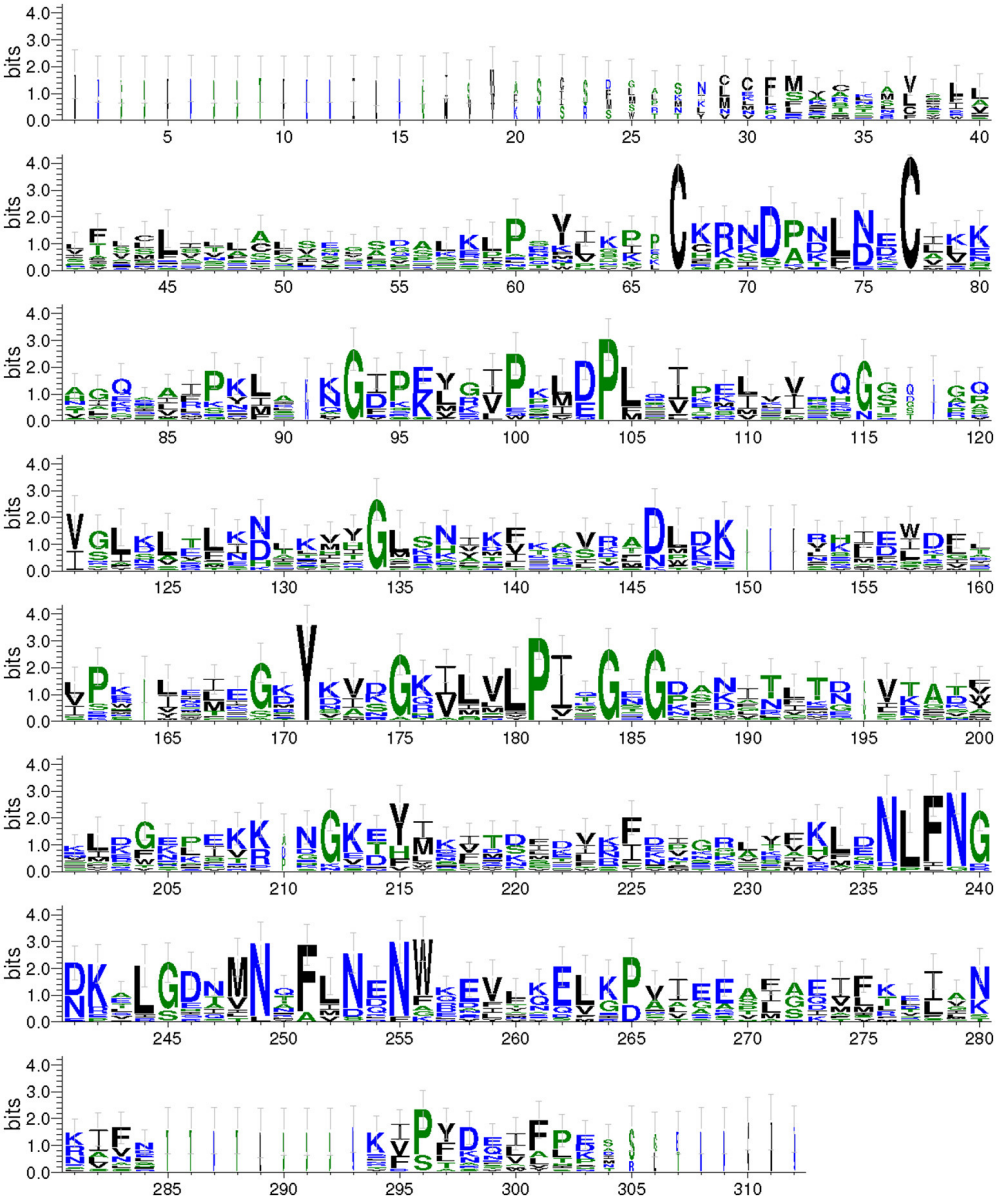
**WebLogo of the Takeout protein sequences**. The data are the alignment of 17 Takeout proteins from *N. lugens*. The *X*-axis represents the amino acid position. The *Y*-axis (bits) represents the relative proportion of the amino acids at one position. The height of the logo varied inversely with the variability at the position.

We aligned the predicted Takeout protein sequences and graphical presentation of the sequence conservation by the overall height (Figure 2). The conserved amino acids were distributed throughout the entire Takeout protein sequence. A comparison of these Takeout proteins revealed two highly conserved cysteine residues (C) at the N terminal, four highly conserved glycine residues (G) and two highly conserved proline residues (F, Figure 2) in the middle of the protein.

### Male biased expressions of brown planthopper *takeout* family genes

**Figure 3:**
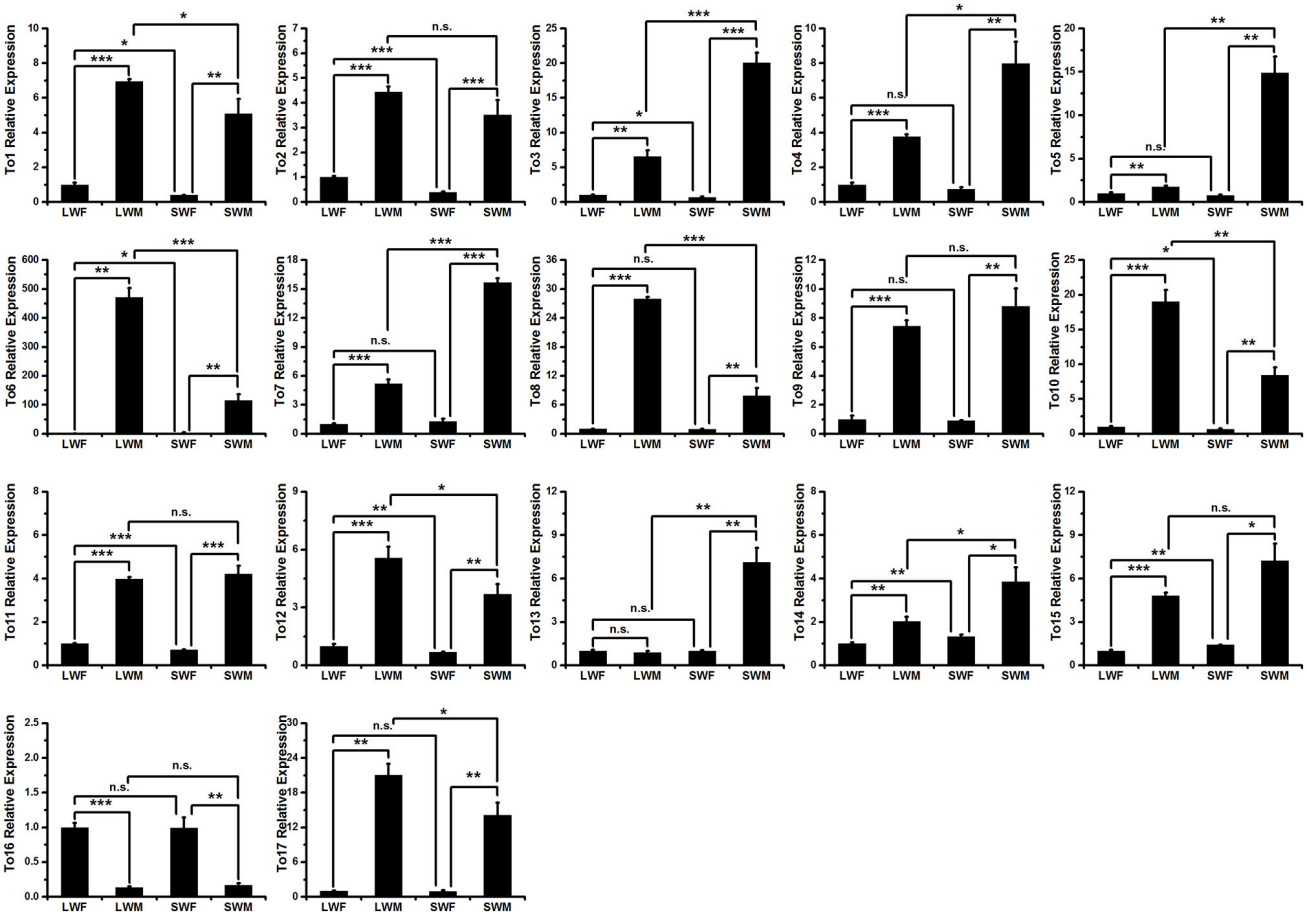
**Expressions of the *takeout* family genes in four types of brown planthopper adults**. LWF, long-wing female; LWM, long-wing male; SWF, short-wing female; SWM, short-wing male. Student's *t*-test was used, ^*^P < 0.05, ^**^P < 0.01, ^***^P < 0.001.

To study whether the expressions of the *takeout* genes in brown planthopper are male biased, we measured the expressions of the *takeout* family genes using qRT-PCR and compared the expressions in males to those in females in the two wing forms. The results showed that the expressions of 16 of the 17 *takeout* genes are male biased (Figure 3), which is consistent with previous studies by Dauwalder et al. in *D. melanogaster* (Dauwalder et al., 2002). However, in contrast, we found one *takeout* gene, *NlTO16*, that was more highly expressed in females than in males (Figure 3), i.e., the fold change is 7.5 times in the long-wing form and 6 times in the short-wing form. The expression of *NlTO16* in the long wing and short wing forms was not significantly different.

### The effect of JH on the expressions of *takeout* family genes

**Figure 4:**
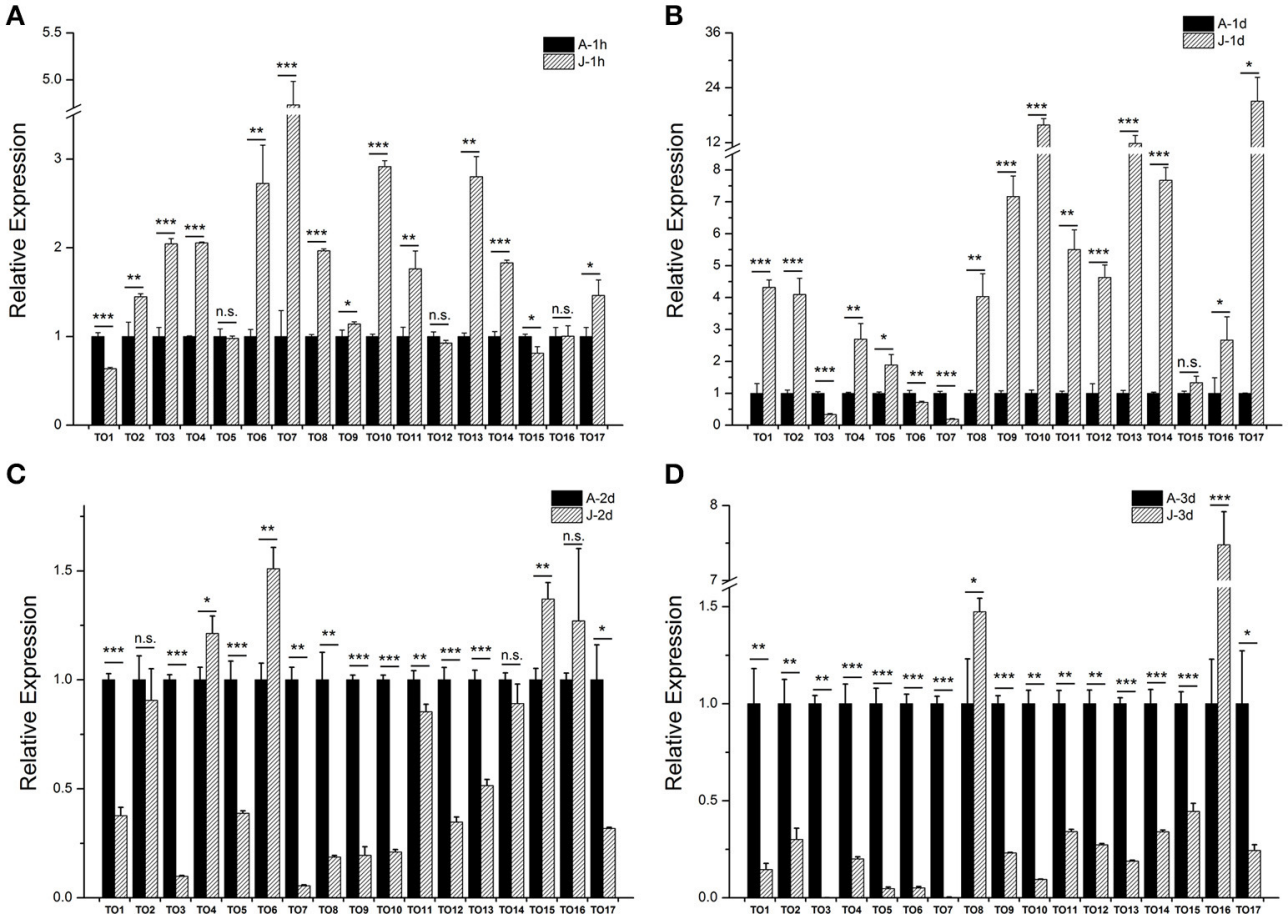
**The effect of juvenile hormone III (JHIII) on the expressions of the *takeout* family genes**. The measurements of the *takeout* family genes were carried out 1 h **(A)**, 1 day **(B)**, 2 days **(C)**, and 3 days **(D)** after the JHIII treatment. The expressions of the *takeout* genes *TO1*-*TO17* after the JHIII treatment were compared with the control nymphs that were treated with acetone. Student's *t*-test was used, ^*^P < 0.05, ^**^P < 0.01, ^***^P < 0.001.

Our previous study showed that the expression of brown planthopper *NlKr-h1* is induced by JH or its mimics (Jin et al., 2014). The expression of *NlKr-h1* was significantly high (≈5 times) 1 day after the JH treatment (Jin et al., 2014). Therefore, we measured the expressions of the *takeout* family genes 1 day after the JHIII treatment (Figure 4). The result showed that 14 of the 17 *takeout* genes are up-regulated, and the expression of 13 *takeout* genes was significantly high 1 day after the JHIII treatment (Figure 4B). However, the expression of 12 *takeout* genes was significantly high 1 h after the JHIII treatment (Figure 4A), and the fold changes are lower than those following a 1 day treatment. The expressions of *NlTO9, 10, 13, 14*, and *17* increased more than 6-fold 1 day after the JHIII treatment. Only three genes, *NlTO 3, 6*, and *7*, are down-regulated after the JHIII treatment and decreased by <6 times 1 day after the JHIII treatment (Figure 4B). This finding is consistent with previous studies in the honey bee *A. mellifera* (Hagai et al., 2007) and the tobacco hornworm *Manduca sexta* (Du et al., 2003) in which the expression of the *takeout* gene is regulated by JH. However, when we measured the expressions of the *takeout* genes 2 and 3 days after the JHIII treatment, the expressions of 9/15 of the 17 genes were significantly reduced (Figures 4C,D).

### The expressions of the *takeout* family genes in met and interacting proteins down-regulated brown planthoppers

**Figure 5:**
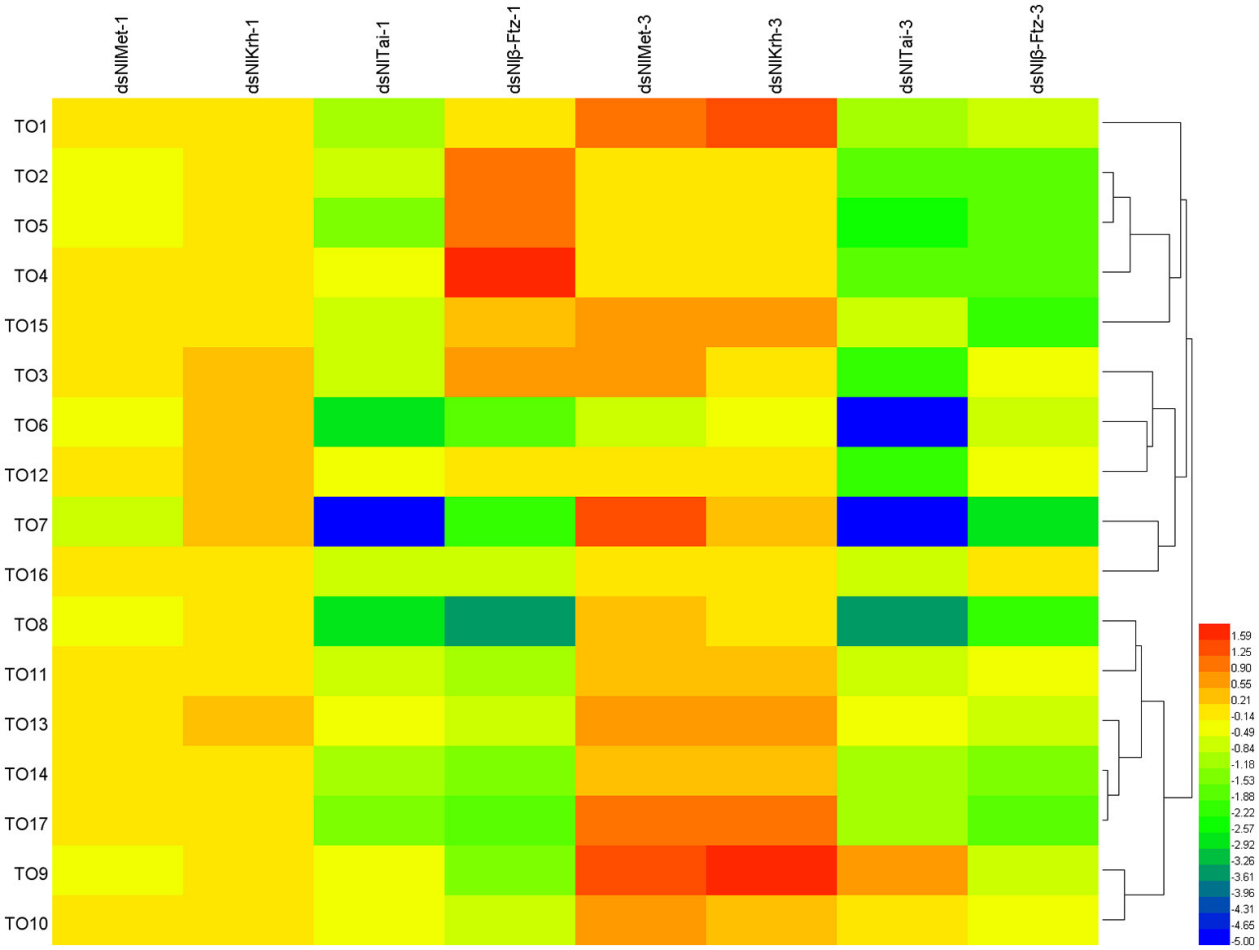
**The expressions of the *takeout* family genes in *NlMet*, *NlKr-h1*, *NlTai*, and β-*Ftz* down-regulated brown planthopper**. The measurements of the *takeout* family genes were carried out 1 and 3 days after the dsRNA injection. The expressions of the *takeout* genes *TO1*–*TO17* after the *NlMet, NlKr-h1, NlTai*, or β*-Ftz* dsRNA injections were compared with the control, which was injected with GFP dsRNA. Heatmap was used for the visualization of the expression changes. *NlMet-1, NlKr-h1-1, NlTai-1*, and β*-Ftz-1* indicate 1 day after the dsRNA injection, and *NlMet-3, NlKr-h1-3, NlTai-3*, and β*-Ftz-3* indicate 3 days after the dsRNA injection.

To further understand the regulatory role of JH signaling in the expressions of the *takeout* family genes, we used RNAi to down-regulate the expressions of the JH receptor or its interacting proteins and then measured the fold changes of the *takeout* family genes 1 and 3 days after the dsRNA injection. The expressions of half of the *takeout* family genes are not changed significantly 1 day after the *NlMet* dsRNA and *NlKr-h1* dsRNA injections. One day after the *NlMet* dsRNA injection, the expressions of 10 *NlTO* genes are not changed significantly. The fold changes of five genes are <2, and the fold changes of the remaining genes are <4 (Figure 5, Table 3). However, 1 day after the *NlKr-h1* dsRNA injection, the expressions of 7 *NlTO* genes are not changed significantly. The fold changes of the eight genes are <2, and fold changes of the remaining genes are <4 (Figure 5, Table 3). Three days after the dsRNA injection, the majority of the *takeout* genes are up-regulated, and only four and two *takeout* genes are down-regulated 1 and 3 days after the injection, respectively (Figure 5, Table 3). In addition, only a few genes showed no significant changes (*NlTO12* for *NlMet* and *NlTO8, 12*, and *16* for *NlKr-h1* dsRNA; Figure 5, Table 3). In summary, the *takeout* family genes showed relatively stable expressions 1 day after the *NlMet* and *NlKr-h1* dsRNA injections, while after 3 days, the expressions of the majority of the *takeout* family genes changed significantly (Figure 5, Table 3).

**Table 3:** **Expression changes of the *takeout* genes by juvenile hormone III (JHIII) treatment and RNAi**.

**Gene**	**JHIII-1**	**dsNlMet-1**	**dsNlKrh-1**	**dsNlTai-1**	**dsNlβ-Ftz-1**	**dsNlMet-3**	**dsNlKrh-3**	**dsNlTai-3**	**dsNlβ-Ftz-3**
*NlTO1*	★★★	★	★	✩✩✩✩✩	n.s.	★★★★★	★★★★★	✩✩✩✩✩	✩✩✩✩
*NlTO2*	★★★	n.s.	n.s.	✩✩✩	★★★★	★	★	✩✩✩✩✩	✩✩✩✩✩
*NlTO3*	✩✩	n.s.	★	✩✩✩	★★	★★	✩	✩✩✩✩✩	✩✩
*NlTO4*	★★	n.s.	n.s.	✩✩	★★★★★	✩	★	✩✩✩✩✩	✩✩✩✩✩
*NlTO5*	★	✩✩	★	✩✩✩✩✩	★★★★★	✩	★	✩✩✩✩✩	✩✩✩✩✩
*NlTO6*	✩	✩	★★	✩✩✩✩✩	✩✩✩✩✩	✩✩✩	✩✩	✩✩✩✩✩	✩✩
*NlTO7*	✩✩✩	✩✩	★★	✩✩✩✩✩	✩✩✩✩✩	★★★★★	★★	✩✩✩✩✩	✩✩✩✩✩
*NlTO8*	★★★	✩	✩	✩✩✩✩✩	✩✩✩✩✩	★	n.s.	✩✩✩✩✩	✩✩✩✩✩
*NlTO9*	★★★★	✩	n.s.	✩✩	✩✩✩✩✩	★★★★★	★★★★★	★★	✩✩✩
*NlTO10*	★★★★★	n.s.	✩	✩	✩✩✩	★★	★★	✩	✩✩
*NlTO11*	★★★	★	n.s.	✩✩✩	✩✩✩✩✩	★★	★	✩✩	✩✩
*NlTO12*	★★★	n.s.	★	✩	n.s.	n.s.	n.s.	✩✩✩✩✩	✩
*NlTO13*	★★★★★	n.s.	★	✩✩	✩✩✩	★★★	★★★★	✩✩	✩✩
*NlTO14*	★★★★	n.s.	n.s.	✩✩✩✩✩	✩✩✩✩✩	★★	★★	✩✩✩✩✩	✩✩✩✩✩
*NlTO15*	n.s.	n.s.	★	✩✩	★★	★★★	★★★	✩✩✩✩	✩✩✩✩✩
*NlTO16*	★★	n.s.	n.s.	✩✩	✩✩	★	n.s.	✩✩✩✩	n.s.
*NlTO17*	★★★★★	n.s.	n.s.	✩✩✩✩✩	✩✩✩✩✩	★★★★★	★★★★★	✩✩✩✩✩	✩✩✩✩✩

*Down-regulated: ✩; Up-regulated: ★;✩/★:1~2-fold;✩✩/★★:2~4-fold;✩✩✩/★★★:4~6-fold;✩✩✩✩/★★★★:6~8-fold;✩✩✩✩✩/★★★★★:>8-fold*.

However, after the injections of *NlTai* and *Nl*β*-Ftz* dsRNA, the expressions of the *takeout* family genes are mainly down-regulated, and the majority of them are significantly different from the control, which was injected with dsGFP (Figure 5, Table 3). The expressions of the *takeout* genes, except for *NlTO9*, are all down-regulated and significantly different after the *NlTai* dsRNA injection (Figure 5, Table 3). One day after the injection, five genes are up-regulated, two genes are not significantly changed, and the remaining genes are down-regulated significantly (Figure 5, Table 3). All genes were down-regulated 3 days after the *Nl*β*-Ftz* dsRNA injection (Figure 5, Table 3). These results indicated that *NlTai* and *Nl*β*-Ftz* are probably more important for maintaining or inducing the expressions of the *takeout* family genes, and *NlMet* and *NlKr-h1* are more important for down-regulating the *takeout* family genes.

## Discussion

Our analysis showed that the brown planthopper *takeout* family genes are conserved across species (Figure 1). However, the functions of these proteins in *N. lugens* are unknown. It is well-documented that JH is involved in the wing polyphenism of the brown planthopper (Iwanaga and Tojo, 1986; Bertuso et al., 2002). The role of *Locusta migraloria takeout* in the behavioral phase change is reminiscent of the role of *takeout* in *N. lugens* because the brown planthopper is polyphenism. Due to the limited knowledge regarding the behavioral phase change in the brown planthopper, determining whether Takeout proteins play a role in this process remains to be explored in the future.

Our experiments showed that the brown planthopper *takeout* family genes are induced 1 h or 1 day after the topical application of JHIII (Figures 4A,B), while the expression levels of most of the *takeout* genes are reduced 2 and 3 days after the JHIII treatment (Figures 4C,D). When we down-regulated the expressions of the JH receptor NlMet and its downstream target NlKr-h1, as well as the NlMet interacting proteins NlTai and Nlβ-Ftz, the expression patterns of the *takeout* family genes are distinct. When *NlMet* and *NlKr-h1* are down-regulated, i.e., 1 day after the dsRNA injection, the expressions of the majority of the *takeout* genes are either not significantly changed or only have slightly changed (Figure 5, Table 3). While after 3 days, the expressions of most of the *takeout* family genes are increased significantly (Figure 5, Table 3). Overall, the effects of the down-regulation of *NlKr-h1* on the expressions of the *takeout* family genes are similar to those of the down-regulation of *NlMet*. However, the down-regulation of the NlMet interacting proteins NlTai and Nlβ-Ftz through RNAi led to a down-regulation of most of the *takeout* family genes 1 and 3 days after the dsRNA injection. This finding indicates distinct roles of NlMet and its interacting proteins in regulating the *takeout* family genes. NlMet and its interacting proteins NlTai and Nlβ-Ftz might act through different mechanisms in regulating the expressions of the *takeout* family genes. As mentioned above, JH could either up-regulate or down-regulate gene expression. In this study, we found that in addition to the crucial role of the JH receptor Met, its interacting proteins NlTai and Nlβ-Ftz also play important roles in regulating the expressions of the *takeout* family genes. However, the roles of *NlTai* and *Nl*β*-Ftz* are distinct from those of *NlMet* in regulating the expressions of the *takeout* genes. This result is consistent with the direct activation of target genes by Met and the repression of target genes with the cooperation of the Hairy/Grouche molecular system (Hagai et al., 2007).

The interaction of Met and Tai in the mosquito *Aedes aegypti* is dependent on JH (Li et al., 2011, 2014). Here, we show that Met and its interacting proteins play distinct roles in regulating the expressions of the *takeout* family genes. Although the expressions of most of the *takeout* family genes significantly increased 3 days after the down-regulation of *NlMet* and its downstream transcription factor *NlKr-h1*, there is only a slight effect 1 day after the dsRNA injection, i.e., the expressions of most of the *takeout* family genes are not significantly changed or only slightly changed.

In the mosquito *Aedes aegypti*, JH activated the phospholipase C (PLC) pathway and protein kinase C (PKC) and immediately increased the levels of inositol 1,4,5-trisphosphate (IP3), diacylglycerol (DAG), and intracellular calcium, thereby activating calcium/calmodulin-dependent protein kinase II (CaMKII; Liu et al., 2015; Ojani et al., 2016). Met protein is phosphorylated upon JH binding (Liu et al., 2015). The increased expressions of the *takeout* genes by the down-regulation of *NlMet* and *NlKr-h1* indicates a possibly distinct mechanism in the regulation of the *takeout* genes by Met and its interacting partners or regulation at different levels, i.e., at the transcriptional, translational or post-translational levels. It is possible that the initial regulation of JH signaling upon ligand binding was affected by the phosphorylation of the Met protein, which leads to the initial unresponsiveness of the *takeout* family genes even though Met transcription was down-regulated, i.e., down-regulating *NlMet* resulted in a change in the phosphorylation of the Met proteins and its down-stream signaling components. Based on our previous study on *Kr-h1*, the genes downstream of JH action are prone to be induced 1 day after the JH application (Jin et al., 2014). In this study, we found that the *takeout* genes are induced 1 h and 1 day after the JHIII application and are reduced 2 and 3 days after the treatment. This result indicates a possible feedback mechanism in regulating the expressions of the *takeout* genes after the induction by JHIII. Additionally, Met and its interacting proteins may act at different developmental stages; in this study, we only tested the expression changes of the *takeout* family genes in brown planthoppers treated at the 5th instar nymph stage. In the future, studies that measure the gene expression levels in other stages and different tissues are to be carried out.

The *takeout* family genes are regulated by JH signaling in *N. lugens*. Although previous studies have shown that *takeout* is involved in feeding and migration, this work advanced our understanding of the molecular function and the regulatory mechanism of JH signaling. Furthermore, this work could help in the development of potential small molecules or the identification of target genes for regulating the expressions of the *takeout* genes behaviors of *N. lugens*, such as feeding and migration, which could be an efficient and environment friendly approach for the control of this pest in the future. The functions of the *takeout* family genes, including its role in polymorphism, remain unclear. Additional experiments are required for the understanding of the mechanisms regulating the *takeout* family genes by JH signaling.

## Author contributions

XL designed the study; LZ and YJ performed the experiment; XL and LZ analyzed the data and wrote the paper.

### Conflict of interest statement

The authors declare that the research was conducted in the absence of any commercial or financial relationships that could be construed as a potential conflict of interest.
